# The long non-coding RNA SNHG1 promotes glioma progression by competitively binding to miR-194 to regulate PHLDA1 expression

**DOI:** 10.1038/s41419-019-1698-7

**Published:** 2019-06-12

**Authors:** Liang Liu, Yan Shi, Jia Shi, Haiyang Wang, Yujing Sheng, Qianqian Jiang, Hua Chen, Xiaojian Li, Jun Dong

**Affiliations:** 1 Department of Neurosurgery, The Second Affiliated Hospital of Soochow University, 1055 Sanxiang Road, Suzhou, 215004 China; 2 Department of Neurosurgery, Nanjing First Hospital, Nanjing Medical University, 68 Changle Road, Nanjing, 210006 China

**Keywords:** Cell biology, Molecular biology

## Abstract

Long non-coding RNAs (lncRNAs) play a vital role in tumourigenesis, including that of glioma. Small nucleolar RNA host gene 1 (SNHG1) is a relatively novel lncRNA that is involved in the development of multiple human tumours. However, its underlying molecular mechanism in glioma has not been completely clarified. In this study, we show that SNHG1 is overexpressed in glioma tissues and cell lines. A series of functional assays suggested that SNHG1 promotes glioma progression in vitro and in vivo. Next, through online databases, a luciferase reporter assay and an RNA pull-down assay, we confirmed that SNHG1 functions as a sponge for miR-194, which acts as a suppressor in glioma. We also verified that pleckstrin homology like domain family A, member 1 (PHLDA1) is the functional target of miR-194. Moreover, rescue experiments demonstrated that SNHG1 regulates PHLDA1 expression in a miR-194-dependent manner. Taken together, our study shows that SNHG1 promotes glioma progression by competitively binding to miR-194 to regulate PHLDA1 expression, which may provide a novel therapeutic strategy for glioma.

## Introduction

Glioma, an important part of neuroma, is the most common primary malignant tumour in the central nervous system and remains a major challenge in the field of neurosurgery^1–3^. At present, there is no cure for glioma, and the average survival time of patients with glioblastoma (GBM) is less than 18 months^4^. The current treatment of glioma, involving surgery, radiotherapy and chemotherapy, has made some progress; however, due to its strong invasion ability and resistance to radiotherapy and chemotherapy, the clinical treatment of glioma remains difficult. Therefore, extensive research on the molecular mechanism of gliomagenesis is urgently needed.

Non-coding RNAs (ncRNAs) are a class of RNAs that do not encode proteins. Numerous studies have shown that ncRNAs have important biological functions^5,6^. Long non-coding RNAs (lncRNAs), an important component of ncRNAs, inactivate or stabilize proteins by regulating the expression of the corresponding gene at the transcriptional and/or posttranscriptional level^7^. A growing number of studies have shown that lncRNA dysregulation is widely present in many types of tumours and is related with multiple biological behaviours of the tumours^8–10^. The lncRNA small nucleolar RNA host gene 1 (SNHG1), located at 11q12.3, has been reported as an oncogene and a prognostic indicator in some human cancers. For example, Gao et al. reported that SNHG1 modulates the growth and metastasis of laryngeal squamous cell carcinoma by regulating YAP1^11^. Cui et al. showed that SNHG1 promotes the progression of pancreatic cancer via the Notch-1 signalling pathway^12^. Another study showed that SNHG1 promotes cell proliferation and invasion and reduces apoptosis in glioma^13^. However, the upstream modulators of SNHG1 and the networks of downstream signalling that bestow the malignant phenotype on glioma cells remain undetermined. Therefore, it is meaningful to explore the underlying molecular mechanism of SNHG1 in glioma.

In the present study, we found that the expression of SNHG1 is upregulated in both glioma tissues and cell lines. The expression level of SNHG1 is correlated with the grade of glioma. Moreover, further analysis revealed that SNHG1 regulates pleckstrin homology like domain family A, member 1 (PHLDA1) expression via the sponging miR-194, leading to the subsequent promotion of glioma cell glucose uptake, proliferation, migration, invasion, angiogenesis and in vivo tumour growth. In conclusion, our study reports, for the first time, that SNHG1/miR-194/PHLDA1 signalling is involved in the progression of glioma, which leads to a more malignant phenotype and may be a novel potential therapeutic target for glioma.

## Results

### SNHG1 expression is upregulated in glioma tissues and cell lines

**Fig. 1:**
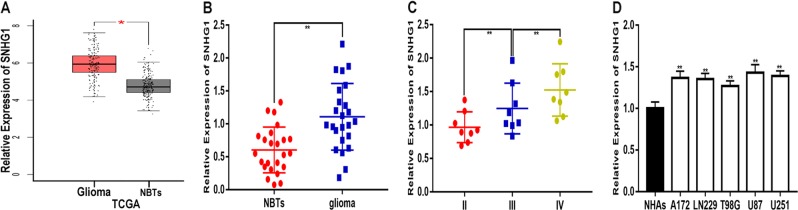
The expression of SNHG1 in glioma tissues and cell lines. **a** The expression level of SNHG1 in TCGA. **b** The expression level of SNHG1 in normal brain tissues (NBTs, *n* = 24) and glioma tissues (glioma, *n* = 24). **c** The expression level of SNHG1 in different grades of glioma. **d** The expression level of SNHG1 in glioma cell lines. **P < 0.01

We initially analysed the expression of SNHG1 in The Cancer Genome Atlas (TCGA) (http://gepia.cancer-pku.cn/index.html) and found that SNHG1 expression was overexpressed in glioma compared to normal brain tissues (NBTs) (*P* < 0.0001) (Fig. 1a). To further verify the results found in TCGA, we explored the expression pattern of SNHG1 in the clinical samples (*n* = 24) collected during surgery. Compared to normal brain tissues, SNHG1 expression was higher in glioma tissues (*P* < 0.0001) (Fig. 1b). We also divided the glioma tissues into three different grades according to the histopathological classification formulated by the World Health Organization (WHO)^14,15^ and found that SNHG1 expression was related to the grade of glioma (Fig. 1c). Next, we detected the expression of SNHG1 in glioma cell lines (A172, LN229, T98G, U87 and U251) and normal human astrocytes (NHAs). SNHG1 was overexpressed in glioma cell lines, especially in U251 and U87 (Fig. 1d). Taken together, these findings indicate that SNHG1 is upregulated in glioma and is associated with the grade of glioma.

### SNHG1 promotes glucose uptake and the proliferation, migration, invasion and angiogenesis of glioma in vitro and tumour growth in vivo

**Fig. 2:**
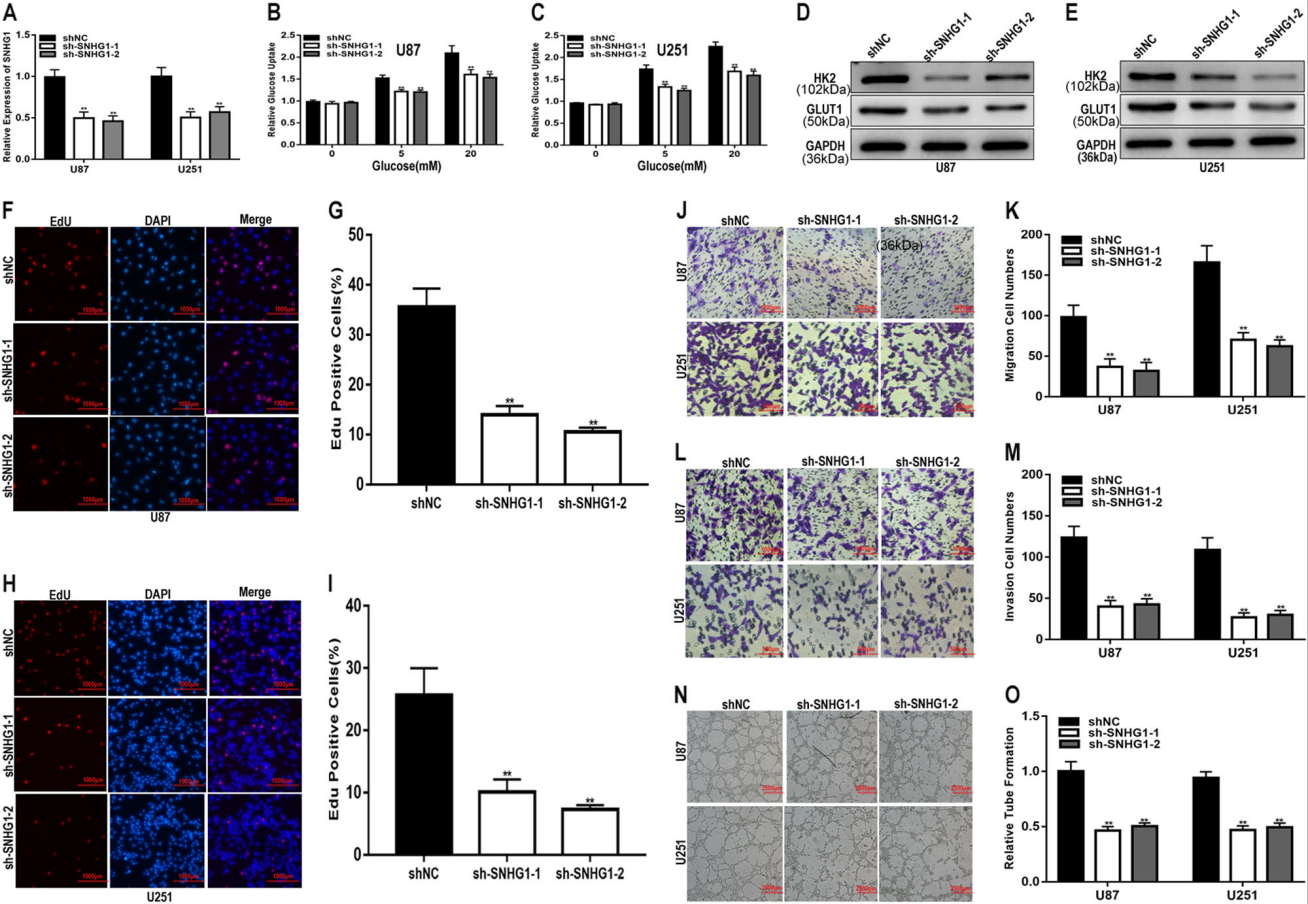
Downregulation of SNHG1 inhibits glucose uptake, proliferation, migration, invasion and angiogenesis of glioma in vitro. **a** qRT-PCR analysis of SNHG1 expression in U87 and U251 cell lines transfected with shNC, sh-SNHG1-1 or sh-SNHG1-2. **b**, **c** Glucose uptake ability was determined after downregulation of SNHG1 in glioma cell lines using a glucose uptake assay. **d**, **e** The expression level of glucose metabolism-related genes was analysed by western blot. **f**–**i** Proliferation capacity was determined after downregulation of SNHG1 in glioma cell lines using EdU assay. **j**, **k** Migration ability was determined after downregulation of SNHG1 in glioma cell lines using a migration assay. **l**, **m** Invasion ability was determined after downregulation of SNHG1 in glioma cell lines using a transwell assay. **n**, **o** Angiogenic capacity was determined after downregulation of SNHG1 in glioma cell lines using an angiogenesis assay. GAPDH used as control. *P < 0.05, **P < 0.01

**Fig. 3:**
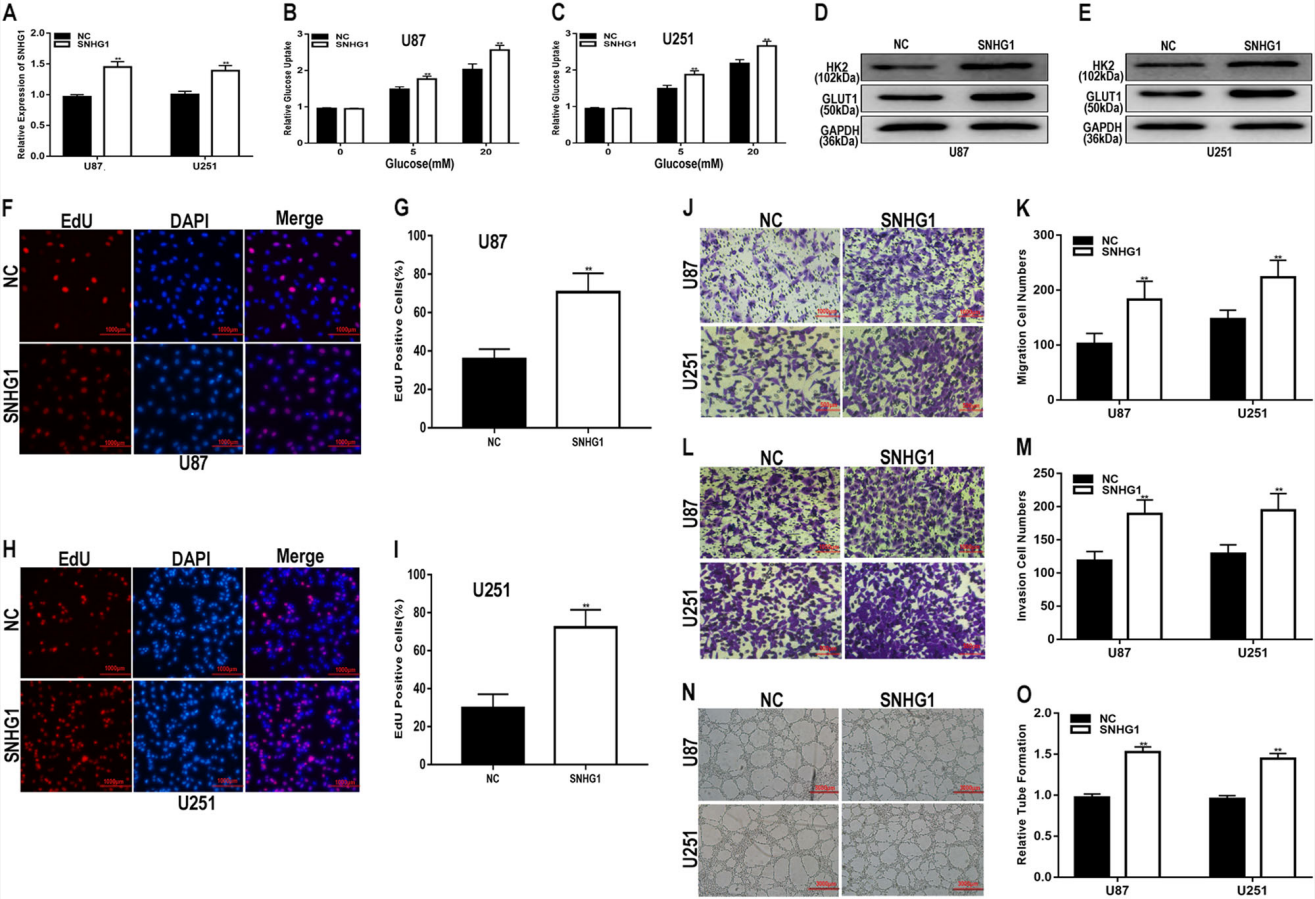
Upregulation of SNHG1 promotes glucose uptake, proliferation, migration, invasion and angiogenesis of glioma in vitro. **a** qRT-PCR analysis of SNHG1 expression in U87 and U251 cell lines transfected with NC or SNHG1. **b**, **c** Glucose uptake ability was determined after upregulation of SNHG1 in glioma cell lines using a glucose uptake assay. **d**, **e** The expression level of glucose metabolism-related genes was analysed by western blot. **f**–**i** Proliferation capacity was determined after upregulation of SNHG1 in glioma cell lines using EdU assay. **j**, **k** Migration ability was determined after upregulation of SNHG1 in glioma cell lines using a migration assay. **l**, **m** Invasion ability was determined after upregulation of SNHG1 in glioma cell lines using a transwell assay. **n**, **o** Angiogenic capacity was determined after upregulation of SNHG1 in glioma cell lines using an angiogenesis assay. GAPDH used as control. *P < 0.05, **P < 0.01

**Fig. 4:**
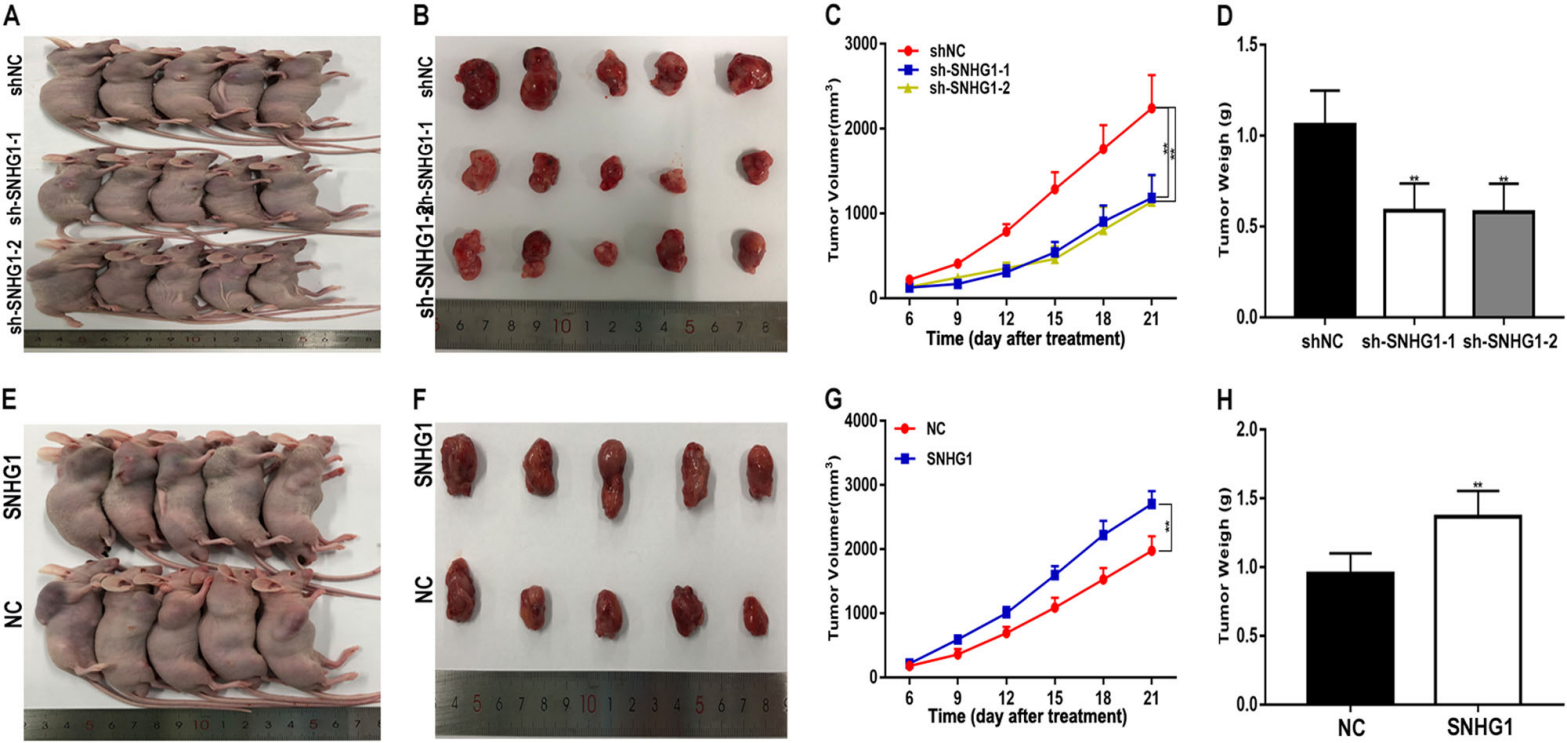
SNHG1 promotes glioma growth in vivo. **a**, **b** Tumour formation was examined in nude mice following the implantation of U87 xenografts with shNC, sh-SNHG1-1 or sh-SNHG1-2. **c**, **d** Tumour weight and volume in the shNC and sh-SNHG1-1 or sh-SNHG1-2 groups. **e**, **f** Nude mice and the excised tumours of U87 xenografts with NC or SNHG1. **g**, **h** Tumour weight and volume in the NC or SNHG1 groups. *P < 0.05, **P < 0.01

To explore the function of SNHG1 in glioma, we transfected glioma cell lines with an shRNA targeting SNHG1 (sh-SNHG1-1 and sh-SNHG1-2), and the transfection efficiency was verified by qRT-PCR (Fig. 2a). Some studies have reported that tumour cell metabolism is reprogrammed to balance biosynthetic processes with energy supply, which is a hallmark of cancer^16^. We aimed to determine whether SNHG1 was associated with glucose metabolism in glioma cell lines. To address our hypothesis, we performed glucose uptake assays and found that SNHG1 downregulation suppressed glucose uptake in glioma cells and attenuated the expression of genes involved in glucose metabolism, including HK2 and GLUT1 ((Fig. 2b–e), whereas the upregulation of SNHG1 showed the opposite results (Fig. 3a–e). These results indicate that SNHG1 is involved in the process of glucose metabolism in glioma. EdU, migration, invasion and angiogenesis assays showed the abilities of sh-SNHG1 to inhibit cell proliferation, migration, invasion and angiogenesis (Fig. 2f–o), while the corresponding abilities were enhanced in glioma cells transfected with the SNHG1 plasmid (Fig. 3f–o). Next, a xenograft tumour assay was performed to explore the role of SNHG1 in vivo. Compared with the control, the weights and volumes of tumours in the SNHG1 downregulation group were decreased (Fig. 4a–d), while the SNHG1 plasmid caused an increase in tumour weight and volume (Fig. 4e–h). Taken together, these results suggest that SNHG1 promotes the progression of glioma both in vitro and in vivo.

### SNHG1 acts as a sponge for miR-194 in glioma

**Fig. 5:**
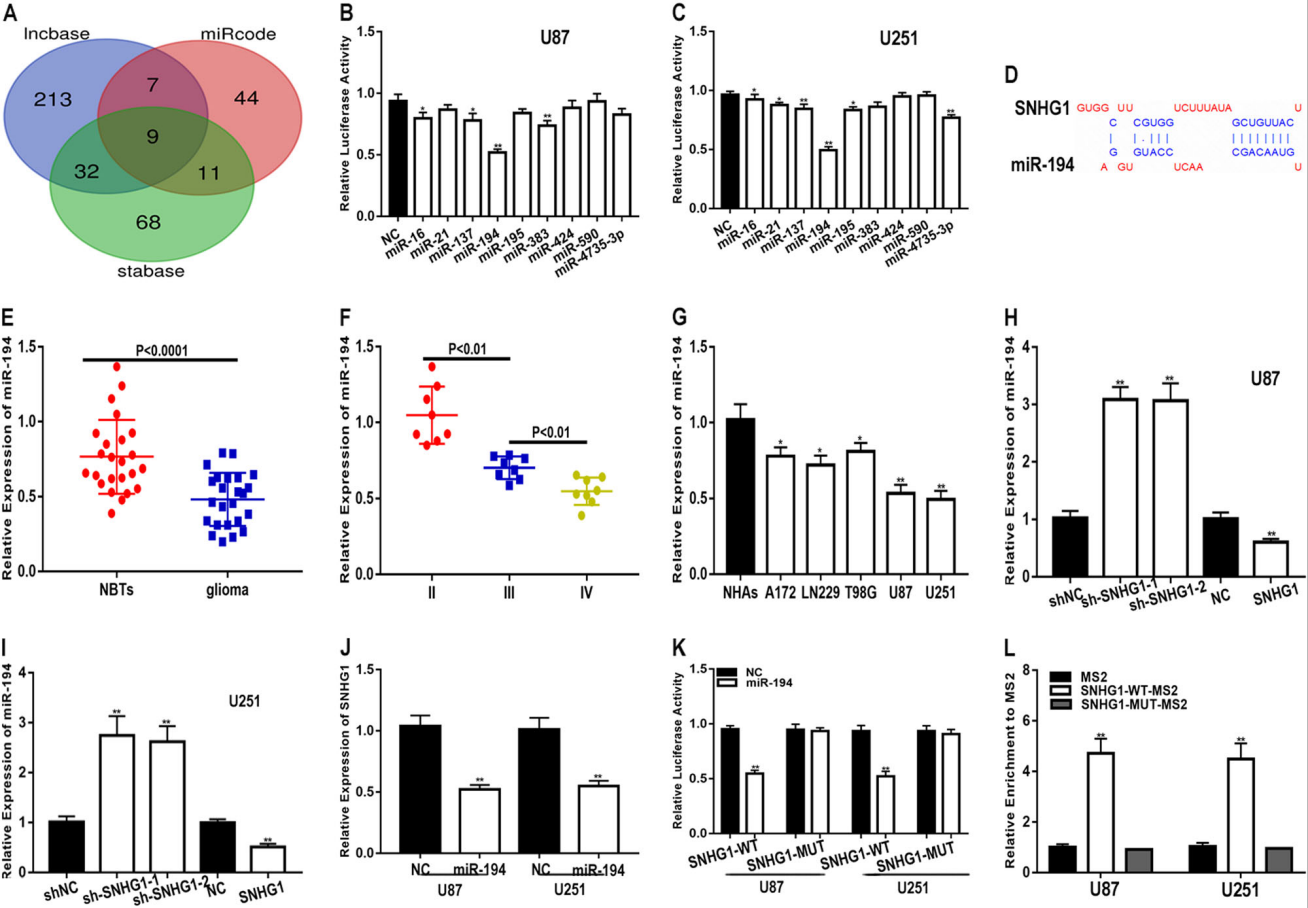
SNHG1 acts as a sponge for miR-194 in glioma. **a** A Venn diagram indicated that nines miRNAs may be biological targets of SNHG1. **b**, **c** Luciferase reporter assays showed that miR-194 dramatically reduced the luciferase activity of SNHG1 luciferase plasmids. **d** Binding sites of miR-194 on SNHG1. **e** The expression level of miR-194 in normal brain tissues and glioma tissues (*n* = 24). **f** The expression level of miR-194 in different grades of glioma. **g** The expression level of miR-194 in glioma cell lines. **h**, **i** qRT-PCR analysis showed that miR-194 was negatively regulated by SNHG1. **j** qRT-PCR analysis showed that SNHG1 was negatively regulated by miR-194. **k** Luciferase reporter assay indicated that miR-194 reduced the luciferase activity of SNHG1-WT rather than SNHG1-MUT. **l** Glioma cells were transfected with MS2-tagged SNHG1-WT, MS2-tagged SNHG1-MUT, or MS2-tagged NC and then assayed by RNA pull down. *P < 0.05, **P < 0.01

Numerous studies have revealed that lncRNAs act as a molecular sponge for miRNAs to regulate tumour progression^17–19^. Inspired by the competing endogenous RNA (ceRNA) mechanism, we speculated whether SNHG1 binds to miRNAs to regulate glioma progression. First, we applied the online database starBase v3.0 (http://starbase.sysu.edu.cn/), miRcode (http://www.mircode.org/) and lncbase predicted v.2 (http://carolina.imis.athena-innovation.gr/) to explore the potential miRNAs that interact with SNHG1. We found that nine miRNAs may be biological targets of SNHG1(Fig. 5a). Next, we performed luciferase reporter assays with the two glioma cell lines (U87 and U251), and the results showed that miR-194 dramatically decreased luciferase activity relative to the other miRNAs (Fig. 5b, c). We performed a bioinformatic analysis using starBase v3.0 and identified the potential binding sites between miR-194 and SNHG1 (Fig. 5d). qRT-PCR revealed that miR-194 is under-expressed in glioma (Fig. 5e). Next, we examined the expression of miR-194 in different grades of glioma and the results showed that the expression level of miR-194 was correlated with the grade of glioma (Fig. 5f). We also evaluated the expression of miR-194 in the six cell lines and the results showed that, compared to NHAs, the expression of miR-194 in glioma cells was downregulated (Fig. 5g). Furthermore, miR-194 was negatively regulated by SNHG1 in U87 and U251 cells (Fig. 5i). Next, we examined the expression of miR-194 in U87 and U251 cells and found that SNHG1 expression was also negatively regulated by miR-194 (Fig. 5j). To further clarify the regulatory relationship between SNHG1 and miR-194, luciferase reporter assays were performed. The luciferase reporter assays revealed that miR-194 significantly decreased the luciferase activity of SNHG1-WT, whereas there was no obvious decrease in luciferase activity in SNHG1-MUT (Fig. 5k). To validate the direct interaction between miR-194 and SNHG1, we performed an RNA pull-down analysis. The precipitated miRNAs were analysed by qRT-PCR. We found that MS2-tagged wild-type SNHG1 (SNHG1-WT-MS2) was significantly enriched for miR-194 in glioma cells compared to the empty vector and SNHG1 with a mutation in the miR-194 binding site (SNHG1-MUT-MS2) (Fig. 5l). These results indicate that SNHG1 acts as a sponge for miR-194 in glioma.

### miR-194 inhibits glioma progression by targeting PHLDA1

**Fig. 6:**
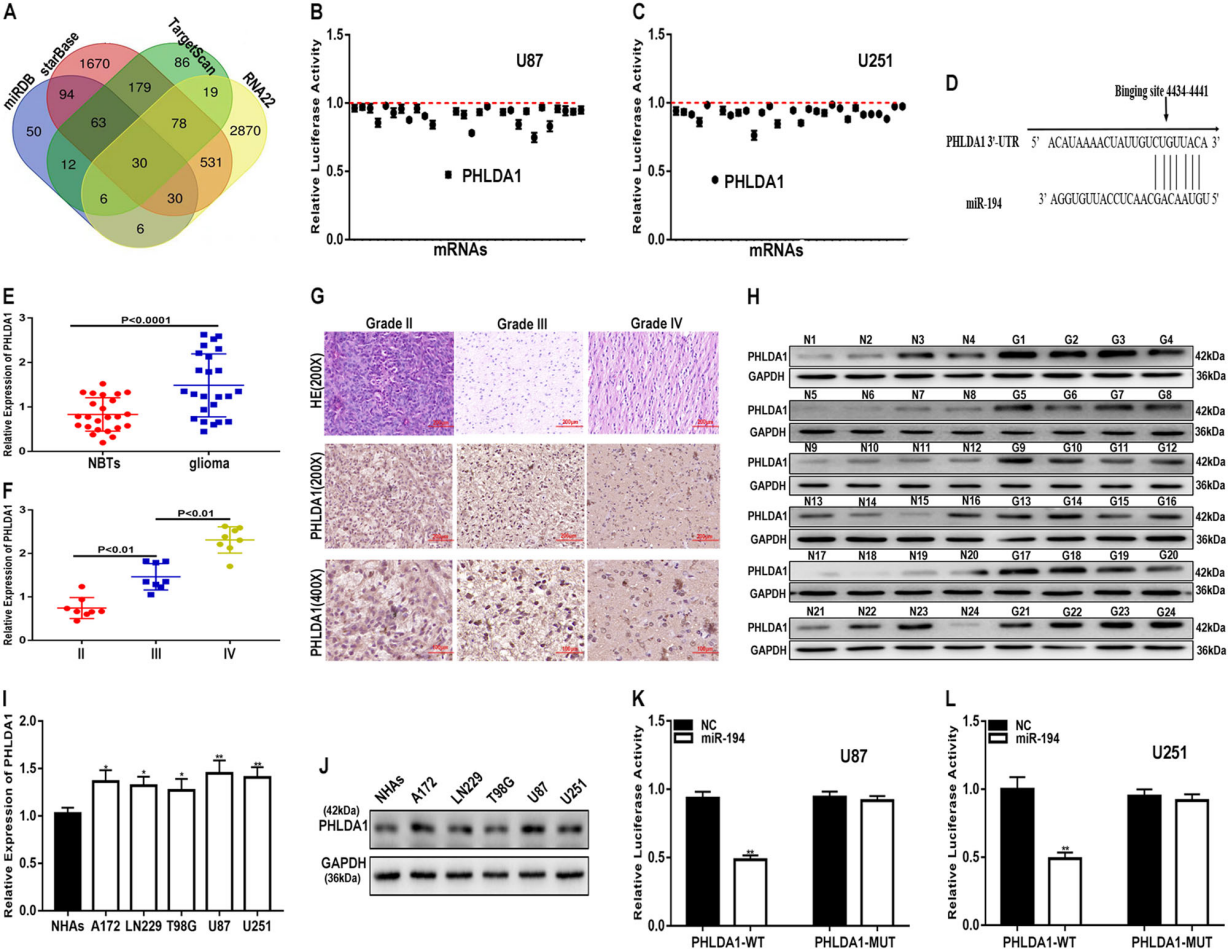
miR-194 inhibits glioma progression by targeting PHLDA1. **a** A Venn diagram indicated that 30 genes may be functional targets of miR-194. **b**, **c** Luciferase reporter assays showed that miR-194 dramatically reduced the luciferase activity of PHLDA1 luciferase plasmids. **d** Binding sites of miR-194 on PHLDA1. **e** The expression level of PHLDA1 in normal brain tissues and glioma tissues (*n* = 24) were measured by qRT-PCR. **f** qRT-PCR analysis of the expression level of PHLDA1 in different grades of glioma. **g** The expression level of PHLDA1 in different grades of glioma detected by immunohistochemistry. **h** The expression level of PHLDA1 in normal brain tissues and glioma tissues (*n* = 24) were measured by western blot. **i**, **j** The expression level of PHLDA1 in normal human astrocyte cells and glioma cell lines was measured by qRT-PCR and western blot analysis. **k**, **l** Luciferase reporter assays indicated that miR-194 reduced the luciferase activity of PHLDA1-WT rather than PHLDA1-MUT. GAPDH used as control. *P < 0.05, **P < 0.01

To confirm the target genes of miR-194, we searched four bioinformatics tools (miRDB, starBase, TargetScan, and RNA22) and jointly predicted that 30 genes may be biological targets of miR-194 (Fig. 6a). Through luciferase reporter assays, we identified PHLDA1 as our research object (Fig. 6b, c). We performed a bioinformatic analysis using TargetScan and identified the potential binding sites between miR-194 and PHLDA1 (Fig. 6d). We evaluated the expression of PHLDA1 in clinical samples by qRT-PCR, immunohistochemistry and western blot analysis. PHLDA1 is overexpressed in glioma and correlates with the grade of glioma (Fig. 6e–h). We detected the expression of PHLDA1 in glioma cell lines as well and the result is consistent with data from the glioma tissues (Fig. 6i, j). To further clarify the relationship between miR-194 and PHLDA1, luciferase reporter assays were performed and the results revealed that miR-194 significantly decreased the luciferase activity of PHLDA1-WT, whereas there was no obvious decrease in luciferase activity in PHLDA1-MUT (Fig. 6k, l). These results suggest that PHLDA1 is the target of miR-194.

**Fig. 7:**
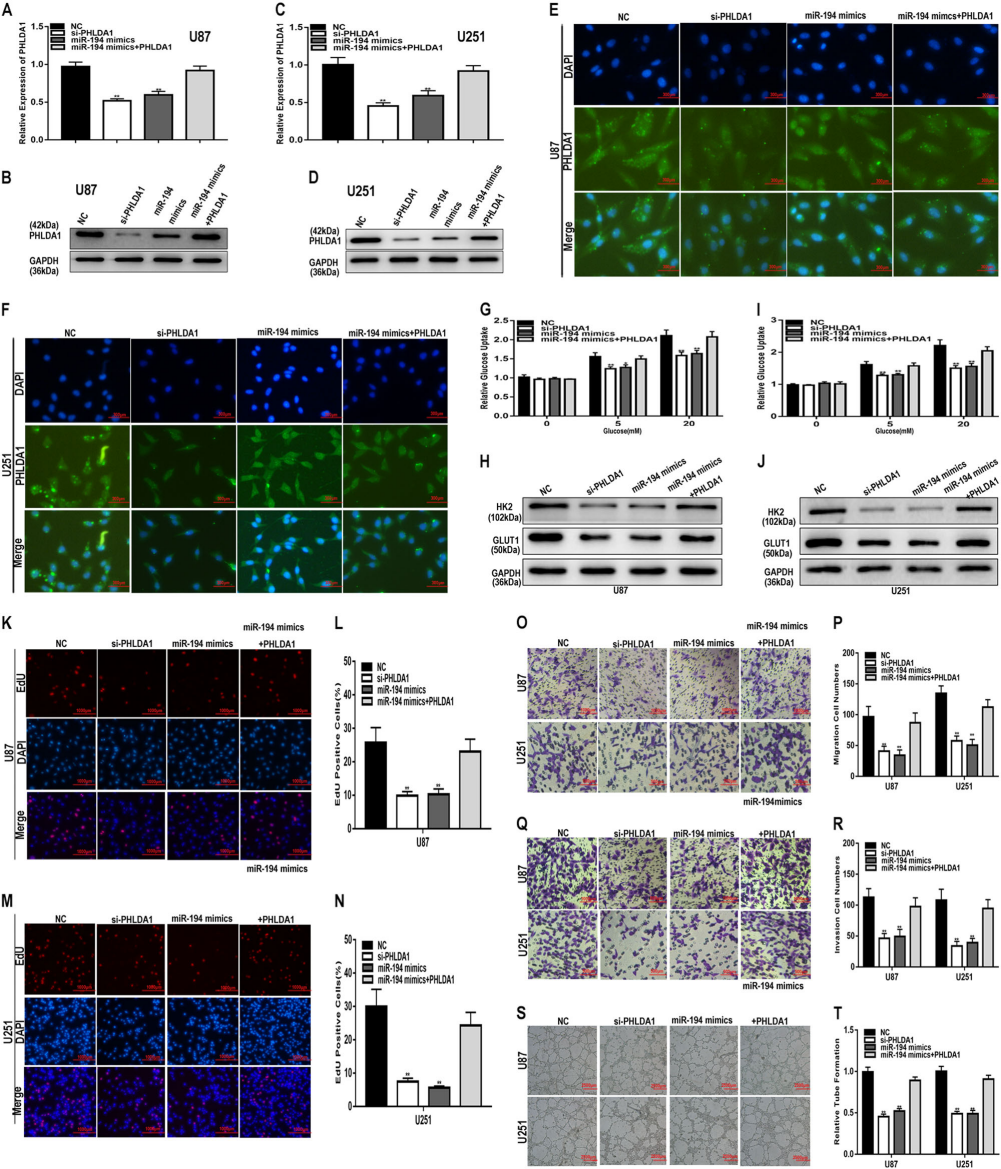
The biological functions of miR-194 and PHLDA1 in glioma. **a**–**f** qRT-PCR, western blot and immunofluorescence analyses of PHLDA1 expression in U87 and U251 cell lines transfected with NC, si-PHLDA1 miR-194 mimics, or miR-194 mimics together with PHLDA1 plasmids. **g**, **i** Glucose uptake ability was determined in U87 and U251 cell lines transfected with NC, si-PHLDA1, miR-194 mimics, or miR-194 mimics together with PHLDA1 plasmids. **h**, **j** The expression level of glucose metabolism-related genes was analysed by western blot. **k**–**n** Proliferation capacity was determined in U87 and U251 cell lines transfected with NC, si-PHLDA1 miR-194 mimics or miR-194 mimics together with PHLDA1 plasmids using EdU assay. **o**, **p** Migration ability was determined in U87 and U251 cell lines transfected with NC, si-PHLDA1, miR-194 mimics or miR-194 mimics together with PHLDA1 plasmids using a migration assay. **q**, **r** Invasion ability was determined in U87 and U251 cell lines transfected with NC, si-PHLDA1, miR-194 mimics or miR-194 mimics together with PHLDA1 plasmids using a transwell assay. **s**, **t** Angiogenic capacity was determined in U87 and U251 cell lines transfected with NC, si-PHLDA1, miR-194 mimics or miR-194 mimics together with PHLDA1 plasmids using an angiogenesis assay. GAPDH used as control. *P < 0.05, **P < 0.01

To verify the regulatory relationship between miR-194 and PHLDA1, we transfected an siRNA against PHLDA1 (si-PHLDA1), miR-194 mimics or the miR-194 mimics together with the PHLDA1 plasmid into U87 and U251 cells. qRT-PCR and western blot analysis showed that the si-PHLDA1 and miR-194 mimics decreased the expression of PHLDA1 and that the inhibitory effect was partly counteracted by the PHLDA1 plasmid (Fig. 7a–d). The immunofluorescence assay yielded results consistent with the results of qRT-PCR and western blot (Fig. 7e, f). In addition, through a series of assays, we obtained some interesting results. According to the glucose uptake assay results, the decreased glucose uptake induced by miR-194 mimics was recovered by transfection with the PHLDA1 plasmid (Fig. 7g–j). The decreased proliferation caused by miR-194 mimics was ameliorated by the PHLDA1 plasmid (Fig. 7k–n). Meanwhile, miR-194 mimics-induced migration and invasion inhibition were reversed by PHLDA1 plasmid (Fig. 7o–r). The inhibitory effect of miR-194 mimics on angiogenesis was reversed by the PHLDA1 plasmid as well (Fig. 7s, t). Thus, we conclude that miR-194 inhibits glioma progression by targeting PHLDA1.

### PHLDA1 promote glioma growth in vivo

**Fig. 8:**
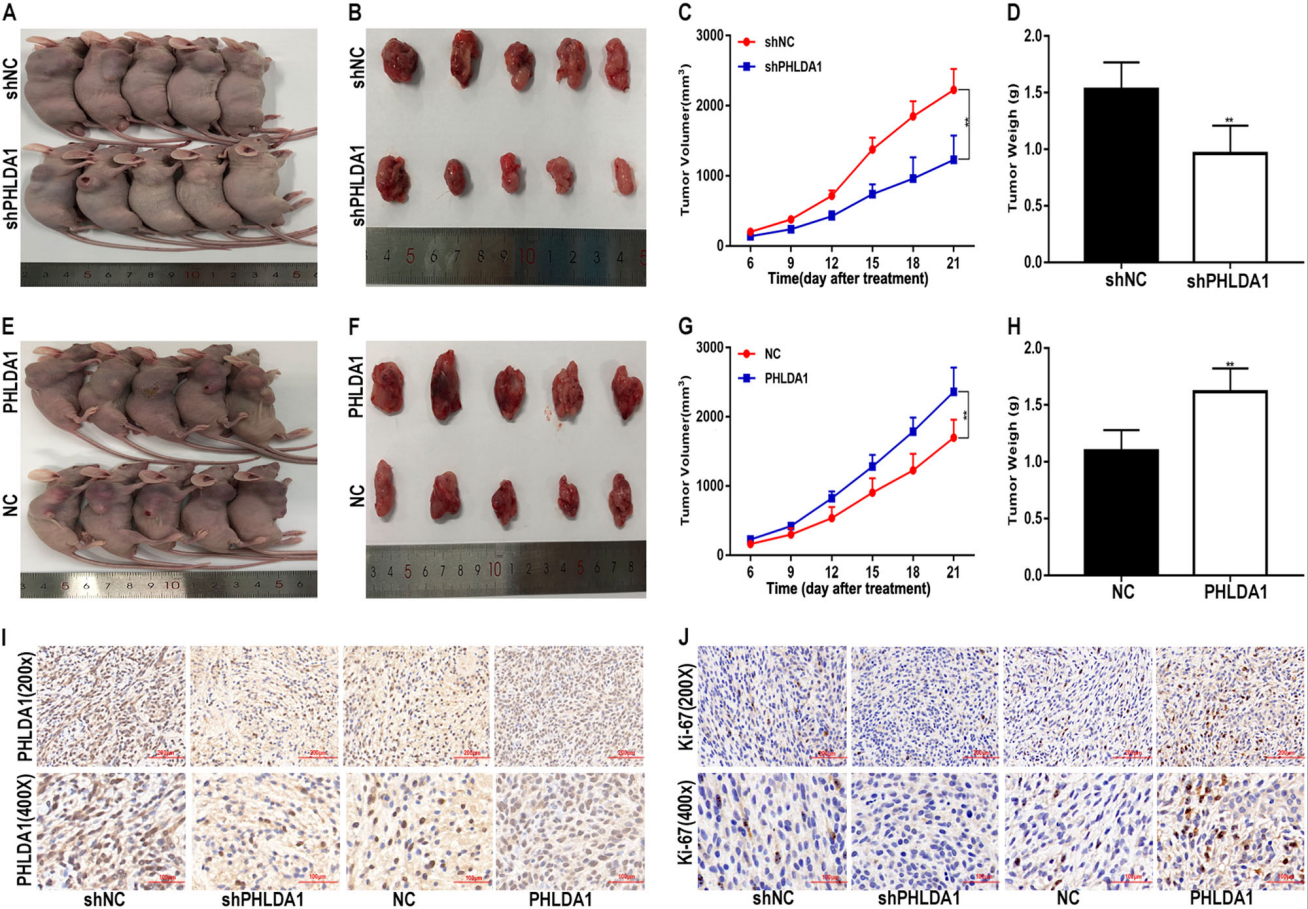
PHLDA1 promotes glioma growth in vivo. **a**, **b** Tumour formation was examined in nude mice following the implantation of U87 xenografts with shNC or sh-PHLDA1. **c**, **d** Tumour weight and volume in the shNC and sh-PHLDA1 groups. **e**, **f** Nude mice and the excised tumours of U87 xenografts with NC or PHLDA1. **g**, **h** Tumour weight and volume in the NC and PHLDA1 groups. **i** Immunohistochemistry for PHLDA1 in the four groups. **j** Immunohistochemistry for Ki-67 in the four groups. *P < 0.05, **P < 0.01

A xenograft tumour assay was performed to explore the role of PHLDA1 in vivo as well. Compared with the control, the weights and volumes of tumours in the PHLDA1 downregulation group were decreased (Fig. 8a–d), while the PHLDA1 plasmid caused an increase in tumour weight and volume (Fig. 8e–h). In additional, the immunohistochemistry for Ki-67 showed that PHLDA1 increased the proportion of Ki-67 positive cells (Fig. 8i, j). Taken together, these results suggest that PHLDA1 promotes the progression of glioma in vivo.

### SNHG1 promotes glioma progression by sponging miR-194 and regulating PHLDA1 expression

**Fig. 9:**
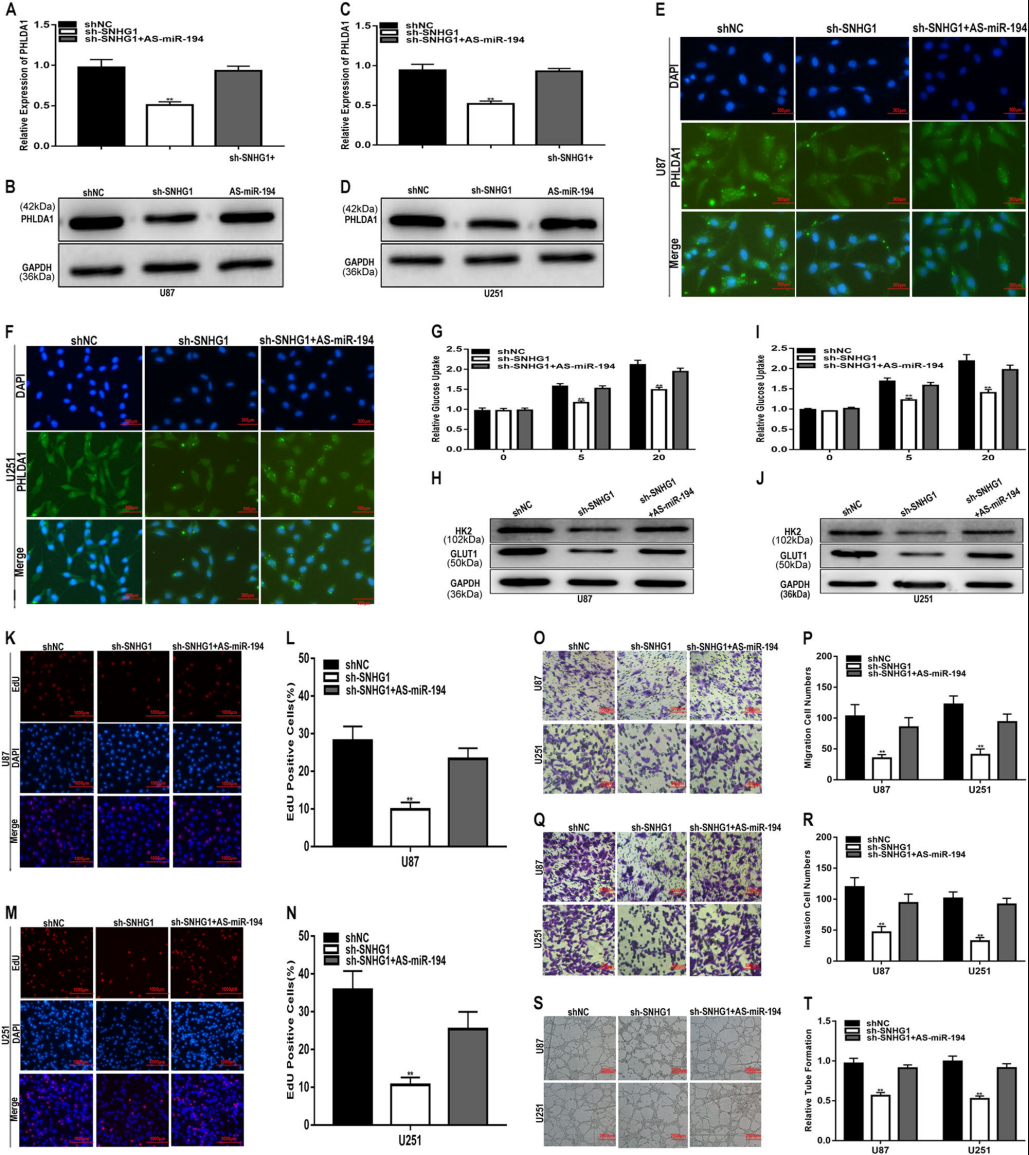
SNHG1 promotes glioma progression by sponging miR-194 and regulating PHLDA1 expression. **a**–**f** qRT-PCR, western blot and immunofluorescence analyses of PHLDA1 expression in U87 and U251 cell lines transfected with NC, sh-SNHG1 or sh-SNHG1 together with miR-194 inhibitors. **g**, **i** Glucose uptake ability was determined in U87 and U251 cell lines transfected with NC, sh-SNHG1 or sh-SNHG1 together with miR-194 inhibitors. **h**, **j** The expression level of glucose metabolism-related genes was analysed by western blot. **k**–**n** Proliferation capacity was determined in U87 and U251 cell lines transfected with NC, sh-SNHG1 or sh-SNHG1 together with miR-194 inhibitors using EdU assay. **o**, **p** Migration ability was determined in U87 and U251 cell lines transfected with NC, sh-SNHG1 or sh-SNHG1 together with miR-194 inhibitors using a migration assay. **q**, **r** Invasion ability was determined in U87 and U251 cell lines transfected with NC, sh-SNHG1 or sh-SNHG1 together with miR-194 inhibitors. **s**, **t** Angiogenic capacity was determined in U87 and U251 cell lines transfected with NC, sh-SNHG1 or sh-SNHG1 together with miR-194 inhibitors using an angiogenesis assay. GAPDH used as control. *P < 0.05, **P < 0.01

The present study suggests that SNHG1 acts as a sponge for miR-194 and that miR-194 targets PHLDA1 to regulate glioma progression. To elucidate the ceRNA mechanism of SNHG1 in glioma, we conducted a series of experiments to explore whether SNHG1 regulates the expression of PHLDA1 in a miR-194-dependent manner. First, we transfected an shRNA against SNHG1 (include sh-SNHG1-1 and sh-SNHG1-2), miR-194 inhibitors or sh-SNHG1 together with miR-194 inhibitors into U87 and U251 cells. qRT-PCR and western blot analysis showed that the sh-SNHG1 downregulated the expression of PHLDA1 and the inhibitory effect was partly abolished by miR-194 inhibitors (Fig. 9a–d). Consistent with the results of qRT-PCR and western blot, immunofluorescence assay indicated the same results (Fig. 9e, f). Moreover, according to the glucose uptake assay results, decreased glucose uptake induced by sh-SNHG1 was recovered by transfection with miR-194 inhibitors (Fig. 9g–j). The decreased proliferation caused by sh-SNHG1 was ameliorated by miR-194 inhibitors (Fig. 9k–n). Meanwhile, the sh-SNHG1-induced inhibition of migration and invasion was reversed by miR-194 inhibitors (Fig. 9o–r). The inhibitory effect of sh-SNHG1 on angiogenesis was reversed by miR-194 inhibitors as well (Fig. 9s, t). Overall, these results suggest that SNHG1 promotes glioma progression by sponging miR-194 and regulating PHLDA1 expression.

## Discussion

In view of the plight of glioma treatment, research on its molecular mechanism is still urgently needed. Numerous recent studies have reported that lncRNAs play a critical role in the development of various tumours, including glioma. For example, Luan et al. showed that lncRNA-H19 promotes glucose metabolism and cell growth in malignant melanoma^20^. Liu et al. reported that lncRNA-HOTAIR acts as a ceRNA to promote glioma progression by sponging miR-126^21^. Huang et al. demonstrated that lncRNA-CDKN2B-AS1 promotes hepatocellular carcinoma growth and metastasis by sponging let-7c-5p^22^. Tycowski et al. found that SNHG1 is required for 18S ribosomal RNA maturation, which is closely related to human tumour progression^23^. In this study, we clarified the mechanism of SNHG1 in the progression of glioma.

Analysis of the differential expression of lncRNAs in TCGA showed that, compared to normal brain tissues, SNHG1 is markedly overexpressed in glioma tissues. SHNG1 is a lncRNA involved in many human diseases, including cancer. For example, Yang et al. reported that SNHG1 alleviates OGD-induced injury in brain microvascular endothelial cells^24^. Zhang et al. found that SNHG1 attenuates cell apoptosis by regulating miR-195 and bcl2-like protein 2 in human cardiomyocytes^25^. Zhang et al. showed that SNHG1 promotes cell proliferation via the PI3K/AKT signalling pathway in pancreatic ductal adenocarcinoma^26^. Yu et al. reported that SNHG1 modulates cholangiocarcinoma by interacting with CDKN1A^27^. There are also studies showed that SNHG1 promotes the progression of glioma, however, the underlying mechanism require further studied^13,28^. In this study, we confirmed the overexpression of SNHG1 in glioma tissues and cell lines. We also demonstrated that SNHG1 promotes glucose uptake and the proliferation, migration, invasion and angiogenesis of glioma in vitro and tumour growth in vivo.

Mounting evidence has shown that lncRNAs act as ceRNAs that sponge miRNAs and reduce the binding of miRNAs to target genes, thereby regulating the expression of the target genes. Previous studies showed that SNHG1 functions as a ceRNA in nasopharyngeal carcinoma^29^, ischaemic stroke^30^ and osteosarcoma^31^. Inspired by these studies, we hypothesized that SNHG1 regulates glioma progression via a ceRNA mechanism. Through a literature search, we found that SNHG1 could bound to miR-154-5p/miR-376b-3p and attenuate its expression, thereby regulating glioma progression^28^. Based on these findings, we wondered whether SNHG1 can adsorb other miRNAs to regulate the progression of glioma. By searching online databases, nine miRNAs were identified as potential targets of SNHG1. Through a series of experiments, including luciferase reporter and RNA pull-down assays, we determined that miR-194 is the functional target of SNHG1. MiR-194 has been proven to be a tumour suppressor in various human cancers. For example, Sun et al. reported that miR-194 is involved in the progression of pancreatic cancer^32^. Li et al. showed that miR-194 has an effect on the epithelial-mesenchymal transition of colorectal adenocarcinoma^33^. In the present study, we concluded that miR-194 inhibits glucose uptake and the proliferation, migration, invasion and angiogenesis of glioma. We also demonstrated that PHLDA1 is the functional target of miR-194. Several studies have shown that PHLDA1 is involved in the development of gastric cancer^34^, ovarian cancer^35^ and papillary adenocarcinoma^36^. However, its role in glioma has not been reported in detail. In the current study, we showed that PHLDA1 is upregulated in glioma and promotes the malignant progress of glioma. We also performed a series of assays and found that the effect of sh-SNHG1 in glioma cells can be partly reversed by miR-194 inhibitors.

In summary, our study shows that SNHG1 is overexpressed in glioma. Functional assays revealed that SNHG1 promotes glioma progression via miR-194 to regulate PHLDA1. Hence, the SNHG1/miR-194/PHLDA1 signalling pathway may be a potential therapeutic target for glioma.

## Materials and methods

### Clinical samples

Twenty-four glioma tissues (obtained from patients with glioma) and normal brain tissues (obtained from patients with brain trauma and treated with internal decompression surgery) were collected from the Department of Neurosurgery, the Second Affiliated Hospital of Soochow University and were immediately stored in liquid nitrogen after surgical resection. No patient received chemotherapy or radiotherapy before surgery. Ethical approval was obtained from the Second Affiliated Hospital of Soochow University. Written informed consent was obtained from all patients.

### Cell culture

All glioma cell lines (U87, U251, A172, T98G and LN229) were purchased from the Chinese Academy of Sciences cell bank (Shanghai, China). The normal human astrocyte cell line was obtained from JENNIO Biological Technology (Guangzhou, China). All the cell lines were cultured in Dulbecco’s Modified Eagle’s Medium (DMEM, Gibco, NY, USA) supplemented with 10% foetal bovine serum (FBS, Sciencell, LA, USA) and were incubated in an atmosphere containing 5% CO_2_ at 37 °C.

### Plasmid construction, oligonucleotides, and cell transfection

The short hairpin RNA (shRNA) targeting SNHG1 (sh-SNHG1-1 and sh-SNHG1-2) and PHLDA1 (sh-PHLDA1), the small interfering RNA targeting PHLDA1 (si-PHLDA1), the overexpression plasmid of SNHG1 (SNHG1), and the corresponding negative control were designed by GenePharma. The sequences are as follows: sh-SNHG1-1, 5′-GCT GAA GTT ACA GGT CT GA-3′; sh-SNHG1-2, 5′-GAC CTA GCT TGT TGC CA AT-3′; SNHG1, sense, 5′-GGG GTA CCG TTC TCA TTT TTC TAC TGC TCG TG-3′ and antisense, 5′-CGG GAT CCA TGT AAT CAA TCA TTT TAT TAT TTT CAT C-3′; the corresponding negative control, 5′-UUC UCC GAA CGU GUC ACG UTT UGC-3′; sh-PHLDA1, forward, 5′-CCG GGA TGG TGC AGT ACA AGA ATC TCG AGA TTC TTG TAC TGC ACC ATC TTT TTG-3′ and reverse, 5′-AAT TCA AAA AGA TGG TGC AGT ACA AGA ATC TCG AGA TTC TTGT ACT GCA CCA TC-3′; si-PHLDA1, 5′-AGG AGC GAT GAT GTA CTG TAA-3′; the corresponding negative control, 5′-TTC TCC GAA CGT GTC ACG TCT-3′.The has-miR-194-5p mimics, inhibitors and corresponding negative control were purchased from GenePharma (Shanghai, China) as well. To construct the SNHG1, miR-194 and PHLDA1 vectors, the full-length sequences of SNHG1 were amplified and inserted into pcDNA3.1 vector (GenePharma, Shanghai, China), and the full open reading frame cDNA clones for miR-194 and PHLDA1 were transcribed. Next, all the products were amplified. Finally, the DNAs were inserted into pcDNA3.1. Oligonucleotides and constructs were transfected into the cell lines using Lipofectamine 3000 (Invitrogen, Carlsbad, CA, USA) according to the manufacturer’s instructions.

### RNA isolation and quantitative real-time polymerase chain reaction (qRT-PCR)

Total RNA from the clinical samples and cell lines was extracted using TRIzol (Invitrogen, Carlsbad, CA, USA). qRT-PCR was used to detect the expression levels of SNHG1, miR-194 and PHLDA1. U6 and GAPDH were used for normalization. The primer sequences are as follows: SNHG1: forward, 5′-ACG TTG GAA CCG AAG AGA GC-3′ and reverse, 5′-GCA GCT GAA TTC CCC AGG AT-3′; miR-194: forward, 5′-GCG GCG GTG TAA CAG CAA CT CC-3′ and reverse, 5′-ATC CAG TGC AGG GTC CGA GG-3′; PHLDA1: forward, 5′-TCA TCC ACA CCA ACT CC AG-3′ and reverse, 5′-ATG CAC TCT TCC CAC TT CC-3′; U6: forward, 5′-CTC GCT TCG GCA GCA CA-3′ and reverse, 5′-AAC GCT TCA CGA ATT TGC GT-3′; GAPDH: forward, 5′-CCA GGT GGT CTC CTC TGA CTT-3′ and reverse, 5′-GTT GCT GTA GCC AAA TTC GTT GT-3′. qRT-PCR was conducted using TaqMan Non-coding RNA Assays and TaqMan miRNA Assays on an ABI Prism 7700 Sequence Detection System (Applied Biosystems, Thermo Fisher Scientific, MA, USA). The 2^–ΔΔCt^ method was used to analyse the data. All samples were run in triplicate.

### RNA pull-down assay

Maltose-binding protein (MBP) affinity purification was used to detect SNHG1-associated miRNAs. The MS2-MBP protein was expressed and purified from *E. coli* following the instructions of the Steitz laboratory^37^. Three bacteriophage MS2 coat protein-binding sites (5′-cgt aca cca tca ggg tac gag cta gcc cat ggc gta caccatcag ggtacgactagtagatctcgtacaccatcagggtacg-3′) were inserted downstream of SNHG1 by site-directed mutagenesis using a Stratagene Quick Change Site-Directed Mutagenesis Kit. To obtain the miRNAs associated with MS2-tagged SNHG1, the glioma cell lines were transfected with MS2-tagged SNHG1 constructs. Ten million cells were used for each immunoprecipitation assay. After 48 h, the cells were subjected to RNA immunoprecipitation analysis as described previously^38^.

### Luciferase reporter assay

The full-length sequence and fragment of SNHG1 that contained the indicated miRNA binding sequences were inserted into pMIR-REPORT vectors. The 3′-UTR fragments of PHLDA1 containing the binding sequence for the specific miRNAs was also inserted into pMIR-REPORT vectors. The glioma cell lines were transfected with the corresponding miRNAs and reporter plasmids. The mutated plasmid was used as a control. Cells were collected 48 h later, and luciferase activity was measured by the Dual Luciferase Reporter Assay System (Promega, Madison, WI, USA).

### Western blot assay

RIPA buffer (KenGEN, shanghai, China) was used to extract total protein from the tissues and cell lines. Protein concentrations were quantified with a BCA Protein Assay Kit (Beyotime, Shanghai, China). The western blot protocols followed were described in our previous study^39^. The primary antibodies used in this study include those against PHLDA1 (1:1000, proteintech, IL, USA), HK2 (1:1000, proteintech, IL, USA), and GLUT1 (1:1000, proteintech, IL, USA). GAPDH (1:1000, YIFEIXUE BIO TECH, Nanjing, Jiangsu, China) was used as a control.

### Glucose uptake assay

Glucose uptake was quantified with a 2-Deoxyglucose Glucose Uptake Assay Kit (Fluorometric, Abcam, CA, USA). Cells were cultured in 96-well plates (1.5 × 10^3^ cells/well) overnight. After treatment with reagents for 24 h, the cells were incubated in the dark (5% CO_2_, 37 °C) with 2-deoxyglucose for 20 min and subjected to the measurement of 2-deoxyglucose uptake on a fluorescence microplate reader (Molecular Devices, CA, USA) at Ex/Em = 535/587 nm^40,41^.

### 5-Ethynyl-20-deoxyuridine (EdU) assay

Cells (20,000) were grown in 96-well plates overnight. 100 µl of EdU (50 µM, RiboBio, Guangzhou, China) was added to each well and cells were incubated for 2 h (5% CO_2_, 37 °C). The cells were then fixed with 0.5% TritonX-100 (KenGEN, shanghai, China.) in PBS (100 µl) for 25 min, and stained with 100 µl Apollo dye solution (RiboBio, Guangzhou, China) for 30 min at room temperature. Next, cell nuclei were stained with DAPI (Invitrogen, Carlsbad, CA, USA) for 30 min. The proportion of cells that incorporated EdU was determined via fluorescence microscopy.

### Transwell migration and invasion assays

Cell migration and invasion capacity were determined by transwell insert chambers (Corning, NY, USA) covered with or without 50 µl of Matrigel (1:8 dilution, BD, NJ, USA). Cells were harvested and dissociated into a single-cell suspension. Next, cells (50,000) in serum-free medium were added to the upper chamber and 500 µl of 20% FBS-containing medium was added to the lower chamber. The chambers were then incubated for 48 h (5% CO_2_, 37 °C). Cells on the upper chamber were scraped and washed away. Simultaneously, cells on the lower chamber were fixed with 4% paraformaldehyde and stained with 1% crystal violet. Cells that underwent migration or invasion were counted in at least three randomly selected microscopic fields^42^.

### Angiogenesis assay

Cell angiogenesis ability was quantified by µ-slide angiogenesis (15-well, ibidi, Germany). The 15-well plates were coated with 10 µl of Matrigel (BD, NJ, USA) and incubated for 2 h to form a layer of Matrigel. Cells were cultured to 90–100% confluence, and the old medium was discarded and replaced with serum-reduced medium (1% FBS) for 24 h. The medium was collected and stored at −80 °C. HUVECs were cultured in basic medium containing 0.2% FBS for 24 h, and the starved HUVECs were trypsinized, collected, counted and resuspended in endothelial cell growth medium supplemented with Low Serum Growth Supplement (LSGS, Gibco, NY, USA). Next, cells were mixed with an equal volume of the conditioned medium and seeded onto the Matrigel-pretreated 15-well plates at 35,000 cells/well. Twelve hours later, tube formation was examined under a light microscope^21^.

### Immunohistochemistry

Immunohistochemistry was performed as described previously^39^. Paraffin-embedded tissues were incubated with a primary antibody against PHLDA1 (1:200, proteintech, IL, USA) or Ki-67 (1:200, CST, MA, USA) overnight at 4 °C, followed by incubation with a biotinylated secondary antibody (1:1000, YIFEIXUE BIO TECH, Nanjing, Jiangsu, China) at room temperature for 1 h. Next, the tissues were incubated with ABC-peroxidase for 1 h, washed three times with PBS, stained with diaminobenzidine for 5 min, and counterstained with haematoxylin. At least three randomly selected visual fields were examined to evaluate the expression of PHLDA1 or Ki-67.

### Immunofluorescence

Cells cultured on a 96-well plate were fixed with 4% paraformaldehyde for 5 min and blocked with 5% goat serum for 2 h. Next, the cells were incubated with a primary antibody against PHLDA1 (1:200, proteintech, IL, USA) overnight at 4 °C, followed by incubation with a FITC-conjugated secondary antibody (1:1000, CST, MA, USA) at room temperature for 1 h. The cells were then washed three times with PBS. At least three randomly selected visual fields were examined to evaluate the expression of PHLDA1.

### Tumour xenograft experiments

Forty-five immunodeficient female nude mice were randomly divided into nine groups. Approximately 2,000,000 logarithmically growing U87 cells stably expressing sh-SNHG1-1, sh-SNHG1-2, SNHG1, sh-PHLDA1, PHLDA1 and corresponding negative control were subcutaneously injected into the nude mice. Three weeks later, the weight and volume of the subcutaneous tumours were quantified (volume was calculated according to the following formula: *V*(mm^3^) = length × width^2^ × 0.5). All animal procedures were approved by the Institutional Animal Care and Use Committee of the Second Affiliated Hospital of Soochow University.

### Statistical analysis

The data are presented as the mean ± standard error and were analysed with SPSS 20.0 (IBM, NY, USA). Statistical evaluation of the data was performed by *t*-test, *q*-test, and one-way-ANOVA. *p*-value of less than 0.05 was considered statistically significant. The results are representative of at least three independent experiments.
